# Addressing missing data in the estimation of time‐varying treatments in comparative effectiveness research

**DOI:** 10.1002/sim.9899

**Published:** 2023-09-19

**Authors:** Juan Segura‐Buisan, Clemence Leyrat, Manuel Gomes

**Affiliations:** ^1^ Centre for Monetary and Financial Studies Madrid Spain; ^2^ Department of Medical Statistics London School of Hygiene and Tropical Medicine London UK; ^3^ Institute of Epidemiology and Health Care University College London London UK

**Keywords:** comparative effectiveness research, inverse probability weighting, missing data, multiple imputation, time‐varying confounding

## Abstract

Comparative effectiveness research is often concerned with evaluating treatment strategies sustained over time, that is, time‐varying treatments. Inverse probability weighting (IPW) is often used to address the time‐varying confounding by re‐weighting the sample according to the probability of treatment receipt at each time point. IPW can also be used to address any missing data by re‐weighting individuals according to the probability of observing the data. The combination of these two distinct sets of weights may lead to inefficient estimates of treatment effects due to potentially highly variable total weights. Alternatively, multiple imputation (MI) can be used to address the missing data by replacing each missing observation with a set of plausible values drawn from the posterior predictive distribution of the missing data given the observed data. Recent studies have compared IPW and MI for addressing the missing data in the evaluation of time‐varying treatments, but they focused on missing confounders and monotone missing data patterns. This article assesses the relative advantages of MI and IPW to address missing data in both outcomes and confounders measured over time, and across monotone and non‐monotone missing data settings. Through a comprehensive simulation study, we find that MI consistently provided low bias and more precise estimates compared to IPW across a wide range of scenarios. We illustrate the implications of method choice in an evaluation of biologic drugs for patients with severe rheumatoid arthritis, using the US National Databank for Rheumatic Diseases, in which 25% of participants had missing health outcomes or time‐varying confounders.

## 1 INTRODUCTION

Routinely collected data from electronic health records, registries and administrative databases are increasingly used in comparative effectiveness research (CER).^1^ For example, regulatory and reimbursement agencies often use these observational data to complement evidence from randomized controlled trials (RCTs) when the latter is limited or inexistent.^2^, ^3^, ^4^ One such area is the evaluation of treatment strategies sustained over time, that is, time‐varying treatments, due to RCT's limitations regarding the short duration of follow‐up and lack of treatment adherence.^5^ A central feature in these studies is that, unlike time‐fixed treatments, treatment status is determined at different points in time. In these settings, patient's progression typically influences future treatments and outcomes, and is itself affected by previous treatments, also known as time‐varying confounding.^6^

Inverse probability treatment weighting (IPTW) is often used to address time‐varying confounding by re‐weighting the sample and balance confounders between comparison groups at each time point. A marginal structural model (MSM), which models the treatment‐outcome relationship over time, can then be applied to the re‐weighted sample to estimate the causal effect of treatment.^7^ In addition to the confounding, non‐randomized studies often have incomplete information on the outcomes of interest and potential confounders over time, which can raise additional challenges for tackling the time‐varying confounding, and magnify the biases if it is not handled appropriately.^6^

When there are missing data in the outcome and/or confounders in studies with time‐varying confounding, inverse probability of missingness weighting (IPMW) is often used to address the missing data by re‐weighting each individual according to the probability of observing the data. This involves estimating two distinct sets of weights, one for the treatment assignment (IPTW) and one for the missing data (IPMW) and combining (multiplying) them to re‐weight the sample. This approach is straightforward to implement when we have monotone missing data patterns, for example when individuals drop out of the study at some point during follow up. However, IPMW may be less practical in settings with non‐monotone missing data patterns, which occur when the missing values appear in an intermittent manner over time across different variables, such as outcomes and confounders. For example, this setting would require specifying separate IPMW for each missing data pattern, which can be challenging when there are many time points and incomplete variables. In addition, the inverse probability weighting (IPW) approach requires estimating and combining different sets of weights for each missing variable, and that may lead to highly variable weights, and ultimately inefficient treatment effect estimates. In this article, we will refer to “IPW” to denote the general statistical approach, and refer to “IPTW” and “IPMW” to denote the use of IPW for treatment assignment and missing data purposes, respectively.

An alternative approach to address the missing data is multiple imputation (MI), which has been widely used in CER studies.^8^, ^9^ The MI approach involves replacing each missing observation with a set of plausible (imputed) values, drawn from the posterior predictive distribution of the missing data given the observed data. A distinct feature of MI is that it allows the missing data to be addressed separately from the confounding adjustments. MI has received far less attention in studies with time‐varying confounding, and its relative merits in this setting are not yet well understood. Previous methodological studies have explored MI and IPW for addressing missing data in studies with time‐varying confounding.^10^, ^11^, ^12^, ^13^ However, their focus has been on missing data in the confounders and monotone missing data patterns, with most studies considering a single data pattern and missing variable. In CER settings, missingness tends to permeate both outcomes and confounders. For example, outcomes are often collected through patient‐reported questionnaires, which are prone to missing data.^8^, ^9^ In addition, missing data patterns may be non‐monotonic, for example patients may fail to complete the questionnaires intermittently over time, or specific items within the questionnaire.^14^, ^15^

This article aims to assess the practical advantages of using MI and IPW to address missing data in both outcomes and confounders, and across both monotone and non‐monotone missing data patterns in the evaluation of time‐varying treatments. For the purposes of this article, and because we consider IPTW for addressing the confounding, the article effectively compares two approaches: (1) the “IPW approach,” which combines IPTW for addressing the confounding with IPMW for addressing the missing data' and (2) the “MI approach,” which combines IPTW for addressing the confounding with MI for handling the missing data.

The remainder of the article is organized as follows. In Section 2, we introduce our motivating example, which will be used to illustrate the methods. Section 3 describes the methods to address the missing data in the time‐varying confounding setting, and presents the design of the simulation study, which assesses the relative performance of the MI and IPW approaches across a wide range of realistic scenarios. Section 4 reports the results of the simulations and those from applying the methods to our case study. Section 5 discusses the findings in light of previous simulation studies and provides directions for further research.

## 2 MOTIVATING EXAMPLE

### 2.1 The US National Databank for Rheumatic Diseases

Biologics are typically more effective and tolerable drugs compared to conventional (non‐biologic) therapies to alleviate symptoms and slow disease progression in patients with rheumatoid arthritis (RA). RCTs have shown that one of the most widely prescribed biologics, etanercept, provides better improvements to patient's health‐related quality of life (QoL) over the short‐term, compared to alternative biologics.^16^ However, there is little randomized evidence about the long‐term effects of etanercept vs other biologics, for example because of the short follow‐up periods as well as the lack of treatment adherence over time.^16^ Our motivating example draws on routinely collected data from the US National Databank for Rheumatic Diseases (FORWARD),^17^ to evaluate the relative effectiveness of etanercept compared to other biologics over the long‐term. FORWARD collects longitudinal data from over 50 000 patients, across 1500 rheumatologists in the US and Canada. It includes rich information about RA patients and the care they receive, collected biannually through either email‐ or post‐based patient‐reported questionnaires.

### 2.2 Causal estimand

The target population in the motivating example is patients with moderate to severe rheumatoid arthritis (disease activity score above 3.2), who fail to respond to conventional disease modifying anti‐rheumatic drugs (eg, methotrexate). The interventions under comparison are “initiation of biologic treatment with etanercept” and “initiation of biologic treatment with any other biologic drug.” Baseline was defined at the point of initiation of biologic, which is clearly recorded by the physician. The follow up period was 6 years, with outcomes, biologic treatment and confounders collected every 6 months. The outcome of interest was patient's QoL, measured using the EQ‐5D questionnaire. The EQ‐5D, which a measure anchored on a scale that includes 0 “death” and 1 “perfect health,” is typically left skewed and includes negative values, which reflect health states judged worse than death. The estimand of interest is the per‐protocol effect of continuous treatment with etanercept vs other biologic drug on the EQ‐5D outcome over a period of 6 years.

### 2.3 Time‐varying confounding

**FIGURE 1:**
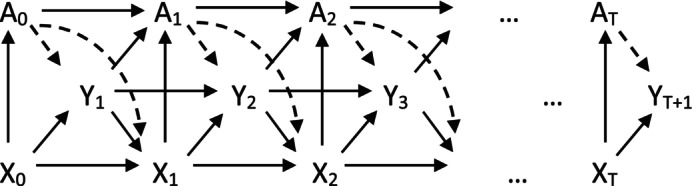
Proposed directed acyclic graph for the causal effect of the etanercept treatment (A) on the EQ‐5D outcome (Y), given the time‐varying confounders (**X**).

The directed acyclic graph in Figure 1 describes the causal effect of treatment ($A_{t}$) on EQ‐5D ($Y_{t}$), with t=0,1,…,T. We assume that there are no direct long‐term effects, although there are long‐term effects mediated through time‐varying confounders ($X_{t}$). In our motivating example, an important time‐varying confounding is the disease activity score (DAS). This index affects the choice of treatment, for example whether to continue or stop the current biologic, and itself is affected by previous biological treatment. At the same time, the DAS is also strongly associated with EQ‐5D, as a higher level of disease activity has a negative impact on QoL dimensions.

We wish to estimate the effect of the dashed arrows, that is, the direct and indirect effects of the etanercept treatment on the EQ‐5D. To improve readability, we assume a simplified causal pathway between treatment and covariates, for example by excluding direct long‐term effects, that is, additional dashed arrows from each $A_{t}$ to $Y_{t+k}$ and $X_{t+k}$, where k>1. In addition, we also omit time‐constant confounders (although these are later considered in both the simulations and case‐study) and assume no effect modification of treatment by time‐varying covariates.

### 2.4 Missing data

Our case‐study nicely illustrates the challenges of simultaneously addressing time‐varying confounding and missing data in both outcomes and confounders. Baseline data is mostly complete in the FORWARD registry as socio‐demographic information and medical history are captured during a baseline “review.” However, follow‐up data, including measurements of time‐varying confounding and outcomes, are collected through online or postal self‐reported questionnaires, which are prone to incompleteness. For example, patients may fail to complete all the items of the EQ‐5D questionnaires or information on time‐varying characteristics (eg, DAS) may not be recorded for all time points.

More specifically, there are three broad types of missing data patterns in the FORWARD registry, exemplified through patient i=1,…,3 in Table 1. In the first pattern, data are intermittently (non‐monotone) missing for either the EQ‐5D or time‐varying confounders. This is a typical setting where patients may omit or not complete some questionnaire items during follow‐up. In the second pattern, patients have non‐monotone missing data in *both* outcome and confounders. For example, these patients may fail to report on prognostic factors and complete their EQ‐5D questionnaires for two or more consecutive time points, but return to have complete data at a later stage. Lastly, there is the usual loss to follow‐up. As with other disease registries, the FORWARD registry is prone to loss to follow‐up, where patients withdraw completely from the registry. We denote this by “monotone missingness due to complete drop‐out.”

**TABLE 1:** The different types of missing data patterns observed in the FORWARD registry.

Patient ID	Time	Treatment	Confounders	Outcomes	Missingness
1	1	A_11_	X_11_	–	Non‐monote missing covariate or outcome (5%)
1	2	A_12_	–	Y_12_	
1	3	A_13_	X_13_	–	
1	4	A_14_	–	Y_14_	
2	1	A_21_	X_21_	Y_21_	Non‐monote missing covariate and outcome (14%)
2	2	A_22_	–	–	
2	3	A_23_	–	–	
2	4	A_24_	X_24_	Y_24_	
3	1	A_31_	X_31_	Y_31_	Monotone due to drop‐out (6%)
3	2	A_32_	X_32_	Y_32_	
3	3	A_33_	–	–	
3	4	A_34_	–	–	

## 3 METHODS

### 3.1 Notation and assumptions

We followed generic notation of the potential outcomes framework in time‐varying settings.^6^, ^7^ We consider a cohort with n individuals, followed up from visit t=0 up to visit t=T. In line with Figure 1, individuals enter the study at baseline (t=0) and receive treatment $A_{0}$ determined by baseline prognostic factors, which include time‐constant covariates (B) and baseline measurements of time‐varying covariates ($X_{0}$). At each visit t, treatment receipt ($A_{t}$) is determined by previous treatment ($A_{t-1}$) and current values of time‐varying confounders ($X_{t}$) and outcome ($Y_{t}$).

For the purposes of this article, we make the following four assumptions to identify causal effects:

1. no interference, which implies that an individual's potential outcome is not affected by the treatment received by another individual. This is usually plausible in non‐communicable disease settings like RA;
2. positivity, which means that all individuals have a probability strictly greater than zero and less than one of receiving treatment at each time point given their prognostic profile. This is plausible in RA settings because it is common for patients to switch between biologics in subsequent time periods due to physician preferences, drug costs, and flexibility to start or stop treatment;
3. consistency, which essentially requires that there are no multiple versions of treatment. This is assumed to hold under an intervention taken “as prescribed by the physician,” particularly amongst a relatively homogenous population with severe disease who are initiating biologics as an add‐on therapy to non‐biologic drugs;
4. no unmeasured confounding, which implies that, conditional on the covariate history, treatment receipt at each visit is independent of unobserved factors. Although we cannot exclude the possibility of residual confounding, FORWARD includes a rich set of time‐constant and time‐varying confounders determining treatment assignment, which increases the plausibility of this assumption.

### 3.2 Marginal structural models

MSMs are a class of models for the estimation of treatment effects in the presence of time‐varying confounding. MSMs model the marginal distribution of the potential outcomes, instead of the observed outcomes, in terms of the treatment values. This article considers MSMs of the following form:

$$E\left(Y^{\bar{a}}\right)=\alpha +\sum_{t=0}^{T}\;\beta_{t}a_{t},$$

where Y is the outcome, $A_{t}$ is the treatment indicator at time t, which takes the value of 1 if the individual is assigned to treatment and 0 otherwise. ${\bar{A}}_{t}$ refers to the treatment history from time point 0 to t. We use capital letters to denote random variables and lower‐case letters to denote their realized values. Thus, we use $\bar{a}\in \bar{A}$ to denote a generic realized treatment history, and $Y^{\bar{a}}$ to denote the potential outcome that would have been observed had the individual received treatment history $\bar{a}$. The coefficients $\beta_{t}$ represent separate treatment effects at each visit, which enables us to estimate long‐term treatment effects. Due to time‐varying confounding, using the observed values to estimate $E\left(Y|\bar{A}=\bar{a}\right)$, for example using standard regression adjustment, would not give an unbiased estimation of $E\left(Y^{\bar{a}}\right)$ because it would block the effect of the treatment on the outcome via the time‐varying confounder.^7^

IPTW can address the time‐varying confounding by reweighting individuals according to the inverse probability of receiving (realized) treatment at each time point, conditional on their covariate and outcome history, $P\left(A_{t}|{\bar{A}}_{t-1},{\bar{Y}}_{t},{\bar{X}}_{t},B\right)$. This works by up‐weighting individuals with low probability of receiving their treatment history. The weights are then the product of the estimated probabilities of the treatment history for t=0,1,2…,T:

$$W_{T}=\prod_{t=0}^{T}\frac{1}{P\left(A_{t}|{\bar{A}}_{t-1},{\bar{Y}}_{t},{\bar{X}}_{t},B\right)}.$$

In the re‐weighted sample, time‐varying (and time‐fixed) confounders are balanced between treated and untreated groups over time, that is, $A_{t}$ is independent of ${\bar{X}}_{t}$ and ${\bar{A}}_{t-1}$. Essentially, IPTW removes all arrows into $A_{0},A_{1},\ldots ,A_{T}$ (Figure 1) but leaves the dashed arrows out of $A_{0},A_{1},\ldots ,A_{T}$ unchanged. To help avoid extremely large weights, for example when the probability of receiving treatment is close to zero, it is recommended to use IPTW with stabilized weights (SW). SW multiply the weights by the probability of receiving treatment conditional on treatment history and baseline covariates $P\left(A_{t}|{\bar{A}}_{t-1},B\right)$, which helps reduce the variability of the weights:

$$SW_{T}=\prod_{t=0}^{T}\frac{P\left(A_{t}|{\bar{A}}_{t-1},B\right)}{P\left(A_{t}|{\bar{A}}_{t-1},{\bar{Y}}_{t},{\bar{X}}_{t},B\right)}.$$

### 3.3 Missing data

Within the MSM framework, IPMW can also be used to address the missing data. The underlying idea is to create weights to balance important confounders between individuals with missing and fully complete data. In practice, IPMW involves estimating a separate model for the probability of observing the data at each time point, computing the missingness weights, and re‐weighting individuals (complete cases) according to inverse of those weights. Then, the missingness weights at time t will be the product of all weights from period 0 to t, which should also be stabilized. The total weights (TW) are then derived by multiplying the missingness weights by the treatment weights:

$${\mathrm{TW}}_{T}=SW_{T}*\prod_{t=0}^{T}\frac{P\left(R_{t}|{\bar{A}}_{t-1},B\right)}{P\left(R_{t}|{\bar{A}}_{t-1},{\bar{Y}}_{t-1},{\bar{X}}_{t-1},B\right)},$$

where $R_{t}$ is the missingness indicator, taking the value of 1 if the individual is observed at time t, 0 otherwise. Re‐weighting the sample using TW will seek to balance observed confounders between treatment groups as well as between individuals with missing and fully observed data. Distinct missingness indicators (and weights) for outcomes ($R_{Y,t}$) and covariates ($R_{X,t}$) will be often required as their missingness patterns are likely to differ. Despite both treatment and missingness weights being stabilized, the TW is likely to be highly variable.

### 3.4 Multiple imputation

The basic idea with MI is to replace the missing observations with a set of plausible values, which are drawn from the posterior distribution of the missing data given the observed data.^18^ Suppose we wish to impute missing values in a normally distributed continuous outcome Y conditional on covariates X. A standard MI procedure using chained equations,^19^ typically using a linear regression, would include the following steps:

*Step 1*—Fit a linear regression of Y on X, using the complete cases, to obtain estimates of coefficients say $\hat{\beta }$ and variance ${\hat{\sigma }}^{2}$ (initial iteration):

$$\left(Y|X,\beta \right)\;˜\;N\left(\beta X,\sigma^{2}\right).$$

*Step 2*—Draw new values of β and $\sigma^{2}$ from the estimated sampling distribution. This essentially adds a perturbation (small change) to allow for the uncertainty in the parameters:

$$\sigma^{2*}˜{\hat{\sigma }}^{2}\left(n_{\mathrm{obs}}-k\right)/g\;\text{and}\;\beta^{*}\;˜\;N\left(\hat{\beta },\sigma^{2*}{\left(X^{T}X\right)}^{-1}\right),$$

where g is a random draw from standard Chi‐2 distribution on $\left(n_{\mathrm{obs}}-k\right)$ degrees of freedom, with $n_{\mathrm{obs}}$ being the number of complete cases and k the number of parameters estimated in the imputation model.

*Step 3*—Fill in the missing values in Y:

$$Y_{\text{miss}}=\beta^{*}X+\epsilon^{*},$$

where $\epsilon^{*}$ is a random draw from $N\left(0,\sigma^{2*}\right)$.

*Step 4*—Repeat steps 2 and 3 M times (number of imputations) to create m=1,2,…,M imputed datasets.

A similar MI procedure applies to discrete outcomes by replacing the linear regression model by an appropriate discrete response model, for example a logistic regression for binary outcome. When the outcome is continuous but not normally distributed, as illustrated by our motivating example, the appropriate imputation approach is less clear. We followed methodological guidance and considered predictive mean matching (PMM),^20^, ^21^ which can relax some of the MI's parametric assumptions, for example about the distribution of the data. PMM uses a similar algorithm as above but in Step 3, instead of drawing the missing values from a normal distribution, it identifies the qth closest individuals (neighbors) who minimize $\left|\beta^{*}X_{h}-\beta^{*}X_{i}\right|,$ where $h=1,\ldots ,n_{\mathrm{obs}}$. Missing outcome values, $Y_{\text{miss}}$, are then imputed from the observed value of one of the qth closest neighbors (chosen at random). Therefore, the distribution of the imputed values should reflect more closely that of the observed values.

Once the data has been imputed, we apply the analysis model (say the IPTW‐based MSM) to each imputed dataset and estimate the parameters of interest, say $\hat{\theta }$ and its variance $V\left(\hat{\theta }\right)$. The resulting estimates obtained across m=1,2,…,M imputed datasets are then combined using Rubin's formulae,^18^ so that the estimates reflect the variability within and across imputations:

$$\hat{\theta }=\sum_{m=1}^{M}{\hat{\theta }}_{m}/M$$

$$V\left(\hat{\theta }\right)=W+\left(1+1/M\right)B,$$

where the total variance is a linear combination of the within‐imputation variance, $W=\sum_{m=1}^{M}V\left({\hat{\theta }}_{m}\right)/M$ (usual variation in the sample), and the between‐imputation variance, $B=\sum_{m=1}^{M}{\left({\hat{\theta }}_{m}-\hat{\theta }\right)}^{2}/\left(M-1\right)$ (proportion of the variation due to the missing data). Unlike the IPW approach, the analysis model is applied to the full sample (observed and imputed), rather than the complete cases only.

### 3.5 Simulation study

The aim of the simulation study is to compare the relative performance of MI and IPW to address monotone and non‐monotone missing data in both outcomes and confounders measured over time. The simulations focus on the estimation of the effect of a time‐varying binary treatment on a continuous outcome, subject to time‐dependent confounding. We follow general recommendations for conducting simulation studies for evaluating statistical methods.^22^

#### 3.5.1 Data generating process

The simulation study is grounded in the motivating example, and considers one continuous outcome, two time‐constant (baseline) confounders (one binary, one continuous), one continuous time‐varying confounder, and one binary treatment, all measured across 5 time periods. The treatment in the initial time period (t=0) is set equal to zero. We initially assume that both outcome and time‐varying confounder are normally distributed, the continuous baseline confounder follows a beta distribution (eg, as age in the case‐study), and the binary covariate is generated such that 6% of the sample had ones (eg, % of smokers). We then expand the simulations to allow a non‐normal (skewed) continuous outcome. We also include additional scenarios with misspecification, by omitting an interaction term, in the imputation and missingness weights models to evaluate the impact on the performance. We follow a recent simulation study^23^ and include an interaction term between two confounders and different degrees of misspecification (“low” vs “high”). Appendix 1 includes the full details about how we modeled misspecification.

**FIGURE 2:**
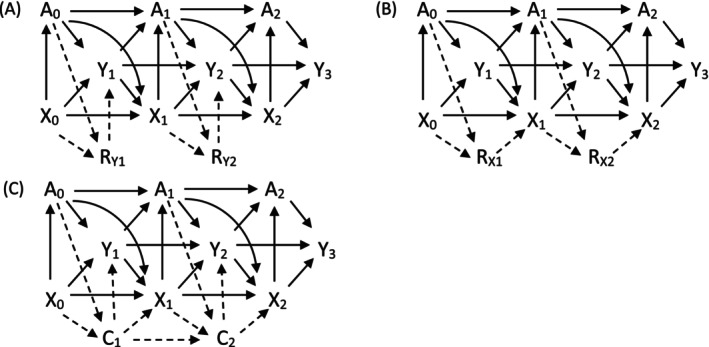
Causal diagrams representing scenarios with different missing data patterns: (A) Non‐monotone missing outcome; (B) non‐monotone missing time‐varying confounder; (C) monotone missing outcome and confounder. A, treatment; X, time‐varying confounder; Y, outcome; R_Y_, missingness indicator for Y; R_X_, missingness indicator for X; C, non‐monotone missingness indicator (attrition/drop‐out).

We simulate a wide range of time‐varying confounding settings with different types of missingness and proportion of missing data informed by the motivating example (Table 1). More specifically, we simulate scenarios with non‐monotone missingness in the outcome or time‐varying confounder (or both). We also simulate scenarios with monotone missingness to reflect settings with complete drop out. Figure 2 describes the data generating mechanism for each of the broad missing data settings (for simplicity, the diagram only presents the first 3 time points). Baseline confounders (B) are omitted from Figure 2 but are associated with $A_{t}$, $X_{t},$ and $Y_{t}$—full details about the data generating process are provided in Appendix 1.

Within each of these broad settings, we consider both low and high proportions of missing data. Scenarios with a high proportion of missing data have 25% non‐monotone missing outcome or time‐varying confounder (or both), and a 25% of monotone missingness. Scenarios with low missingness have 10% non‐monotone missingness and 10% of monotone missing data (see Appendix 2 for full details). We assume data are missing at random (MAR) across all scenarios, that is, missing outcome and confounder data are independent of unobserved values conditional on the observed data, such as past values of treatment receipt, outcome and confounders. Because our simulation study allows for both missing outcomes and confounders, we do not use current values in the missing data generating mechanism as outcomes and confounders may be missing simultaneously.

The estimand of interest is the mean difference in the outcome Y (average treatment effect [ATE]), between the two treatment strategies: “always treat” vs “never treat” at the end of the follow‐up.

#### 3.5.2 Methods

For each scenario, we simulate 1000 datasets with 5000 individuals and 5 follow up visits for each individual. The missing data are analysed using the MI and IPW approach. IPTW weights are computed after regressing treatment on the past treatment, outcome, confounder, and baseline covariates. The IPMW model includes past values of treatment receipt and confounders as per data generating process. All treatment and missingness weights are stabilized and then multiplied for each individual. Due to the limitations of the IPW approach for addressing *non‐monotone* missing data, our implementation of this approach assumes that individuals were censored after the first missing observation, irrespective of whether the missingness was truly monotone (eg, drop‐out) or non‐monotone (eg, intermittent missing observation).

For the MI (via chained equations), we use linear regression to impute the continuous variables and logistic regression for the discrete variables. We consider PMM for scenarios with a skewed outcome. We consider *M* = 50 imputations across all scenarios. We follow previous methodological guidance^24^ to appropriately combine MI with IPTW‐based MSMs. The imputation models include the same variables, that is, past values of treatment receipt and confounders, as the IPMW models to ensure comparability between the IPW and MI approaches.

#### 3.5.3 Performance measures

Statistical performance is assessed according to bias, root mean square error (rMSE) and empirical SE for estimated ATE. The “true” ATE is set to 0.2 across all scenarios. The SEs of treatment effect in our simulation are directly estimated from the MSM and may be somewhat overestimated. However, this applies equally to both IPW and MI approaches and it is not expected to impact their relative performance. While non‐parametric Bootstrap is often recommended to compute SEs, this is too computationally demanding for use in simulations. All analyses are performed using Stata/SE 16.0.

## 4 RESULTS

### 4.1 Simulations

**FIGURE 3:**
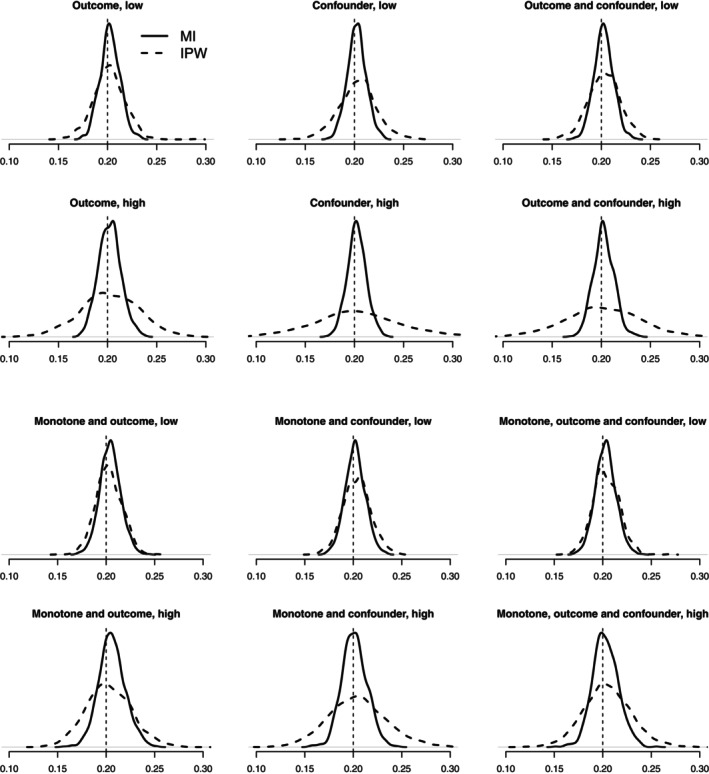
Kernel density plots showing the distribution of ATE estimates using inverse probability weighting (IPW) and multiple imputation (MI). These scenarios assumed a normally distributed continuous outcome but differed according to whether there was non‐monotone (outcome, confounder, or both) or monotone missing data, across low and high levels of missingness. The dashed vertical line reflects the “true” long‐term treatment effect (0.2).

Figure 3 presents Kernel density plots showing the results of the scenarios assuming a normally distributed outcome. Full results can be found in Appendix 3 (Table A2). Overall, both IPW and MI provided unbiased estimates of the long‐term treatment effect. The ATE estimates were within 3% of the “true” values across all scenarios. MI reports visibly lower variability in the ATE estimates (narrower distributions) compared to IPW, with the former providing the lowest rMSE across all scenarios (see Table A2, Appendix 3). The performance of the IPW approach appeared to deteriorate considerably in terms of precision in scenarios with high proportions of missing data and non‐monotone missingness. The rMSE of the IPW was about twice as large (or greater) compared to that of the MI. We replicated these 12 scenarios with a smaller sample size of 1000 individuals, and reported the results in Table A3 (Appendix 3). Both MI and IPW still provided unbiased results, but their rMSE was considerably higher. Overall, the relative gains of MI over IPW were maintained with modest sample sizes, as weighted estimators tend to be inefficient in small samples.

**FIGURE 4:**
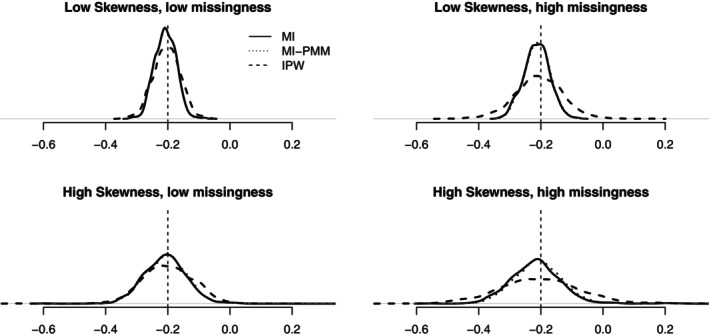
ATE estimates using inverse probability weighting (IPW), multiple imputation using linear regression (MI) and multiple imputation using predictive mean matching (MI‐PMM), for scenarios with a right‐skewed distributed continuous outcome. All scenarios included both monotone and non‐monotone missing data, but differed according to low vs high skewness levels, and low vs high proportions of missing data. The dashed vertical line represents the “true” treatment effect (−0.2).

The results for the scenarios with a skewed outcome are summarized in Figure 4 and Table A4 (Appendix 3) and suggest that MI still provides the lowest rMSE across the scenarios considered. MI using linear regression performs slightly worse in terms of biases compared to the IPW, but still within 8% of “true” values (Table A4, Appendix 3). This is because the standard MI is imputing from the incorrect predictive posterior distribution. MI using PMM reduces the biases and slightly improves precision compared to MI using linear regression; the Kernel density lines mostly overlap between the two MI approaches. Overall, the relative advantage of MI vs IPW is smaller in scenarios with a skewed outcome compared to those with a normally distributed outcome.

The results for the scenarios with misspecification are available in Appendix 4. Overall, the performance of both methods deteriorated under misspecification. For example, biases for the MI went from around 1% to 4%. The relative gains of MI vs IPW decreased across these scenarios, but rMSE was still lower for the former approach.

### 4.2 Case study

Table 2 provides an overview of the baseline patient characteristics (both time‐constant and time‐varying) for the etanercept and comparator groups. The sample considered in this study included 13 002 observations from 1600 patients with rheumatoid arthritis, from January 2011 to April 2017. Similar to RCTs in this area, we restricted our sample to patients with severe disease, who failed to respond to first‐line treatment with methotrexate (non‐biologic). These patients typically switch between different biologics, that is, treatment varies over time, depending on disease progression.

**TABLE 2:** Baseline socio‐demographics and clinical characteristics of patients who initiated etanercept vs other biologics.

Patient characteristics	Etanercept N = 482	Other biologic N = 1118	Standardized mean differences
Time‐constant			
Age, years	58.02 (11.26)	61.75 (11.76)	0.23
Female, %	413 (85.68)	949 (84.88)	0.02
White, %	449 (93.15)	1039 (92.93)	0.01
Graduated from college, %	225 (46.68)	510 (45.61)	0.02
Time‐varying			
Total income ($/year)	65 135 (35 751)	59 624 (34 016)	0.11
Smoking status, %			
Now	31 (6.47)	73 (6.60)	0.02
In the past	182 (38.00)	432 (39.06)	
Never	266 (55.53)	601 (54.35)	
Pain scale, 0–10	3.60 (2.63)	3.75 (2.68)	0.04
HAQ score, 0–3	1.04 (0.71)	1.10 (0.72)	0.06
PAS, 0‐10, %			
< 3.7	268 (55.60)	580 (51.88)	0.05
≥ 3.7	214 (44.40)	538 (48.12)	
DAS, 0–10, %			0.05
< 3.2	285 (59.13)	622 (55.64)	
≥ 3.2	197 (40.87)	496 (44.36)	
Comorbidity score, 0–4	1.77 (1.36)	1.85 (1.34)	0.04
Baseline EQ‐5D	0.742 (0.147)	0.728 (0.154)	0.07

We included the following time‐constant confounders: age, sex, ethnicity (1 if white, 0 otherwise) and education (1 if graduated from college, 0 otherwise). The time‐varying confounders were the pain scale index, HAQ disability index, comorbidity index, patient activity scale (PAS—severity of RA symptoms), disease activity score (DAS—severity disease activity), total annual income and smoking status (never, before and currently smoking). On average, RA patients who initiated etanercept were younger, had higher income, slightly lower disability/pain scores, and better quality of life, compared to those who initiated another biologic.

We estimated the propensity scores by regressing treatment receipt on past treatment, outcome, time‐constant confounders and time‐varying confounders using pooled logistic regression. We then estimated the substantive model by applying a MSM to the reweighted sample, in line with the simulation study. The 95% confidence intervals (CI) were obtained using non‐parametric Bootstrap (1000 bootstrap samples).

**FIGURE 5:**
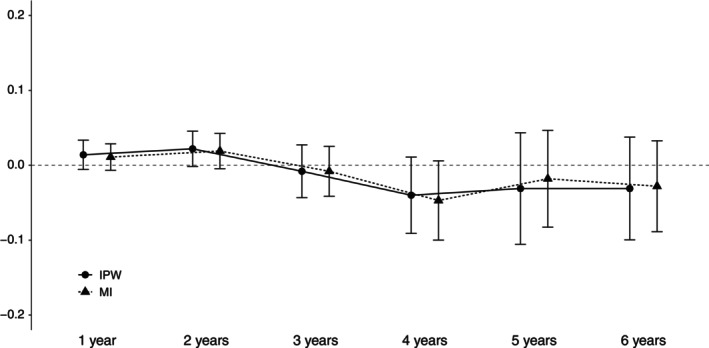
Mean and 95% confidence interval of average treatment effect on EQ‐5D of sustained etanercept treatment over the 6‐year period. MI, multiple imputation; IPW, inverse probability weighting.

Figure 5 reports the cumulative ATE of taking etanercept vs never taking at each time point. The differences in the trajectory of ATE over time between MI and IPW are small. Overall, both approaches suggest that RA patients who initiate etanercept and take it continuously for 6 years does not lead to better health‐related QoL, compared to sustained treatment with other biologics; 95% CI of mean differences in EQ‐5D, both in the short and long‐term, crossed zero. The MI approach reports somewhat more precise (narrower 95% CI) estimates compared to IPW, particularly when there are larger proportions of missing data after year 4.

## 5 DISCUSSION

CER studies that use longitudinal, observational data are likely to be affected by time‐varying confounding and missing data.^10^, ^11^, ^12^, ^13^ Appropriately addressing these two major issues is a necessary step for obtaining unbiased ATE estimates. Considerable attention has been devoted to handling each of these problems separately, but addressing them jointly has received far less attention. Most studies focus on complete case analysis or last observation carried forward, which often result in poor control for the confounding and magnify the biases.^25^, ^26^ Here, we have assessed and illustrated the application of IPW and MI to address missingness in both outcome and time‐varying confounder, across monotone and non‐monotone missing data settings in the evaluation of time‐varying treatments. The simulation study found that both IPW and MI provided mostly unbiased estimates of sustained treatment effects across all scenarios considered. MI consistently provided lower root mean squared error compared to IPW, particularly in settings with larger proportions of missing data and non‐monotone missingness.

This article adds to the existing literature in several ways. First, our work considers MI and IPW in settings with time‐varying treatments, which has additional implications for the choice of method, compared to settings with point treatments.^27^ For example, IPW is often used for tackling confounding over time in addition to the missing data. IPW is most appealing when the model for the weights is relatively simple, and having to combine weights for the confounding and missing data complicates the weighting process considerably.

Second, this article addresses missing data in both outcomes and confounders. Previous studies addressing missing data in time‐varying settings have focused on missingness in the confounders.^10^, ^11^, ^12^, ^13^ As with our study, those studies suggested that both IPW and MI provide minimal biases in estimating time‐varying treatment effects. However, by addressing missingness in outcomes, our article finds that IPW is considerably more inefficient than MI than that suggested by the previous studies. This is because addressing missing outcome data (in addition to missing confounders) requires another set of weights to be estimated for the IPW approach, which leads to more unstable final combined weights, and more variable treatment effects.

Third, missing data in the outcome leads to added complexity for the MI approach. Motivated by the case study, we considered scenarios where the continuous outcome exhibited a non‐normal distributions. Using standard MI methods led to somewhat biased estimates of treatment effects, while the IPW approach still provided minimal biases. Using MI with PMM led to lower biases and improved the precision of the estimates. Overall, our article shows that the relative gains of MI over IPW were smaller in scenarios with skewed data, compared to scenarios with normally distributed outcome.

Fourth, our work expands on existing literature by demonstrating the relative advantages of MI for addressing monotone as well as non‐monotone missing data. The latter type has received little attention in previous studies^11^, ^12^, ^13^, ^24^ but it is typically found in CER studies.^8^, ^9^ For example, outcomes of prime interest to decision makers, such as QoL, are often collected over time using patient‐reported questionnaires, which are completed intermittently. Our simulation study suggested that IPW performed particularly poorly in terms of precision compared to MI in scenarios with non‐monotone missing data. This is largely driven by the inadequacy of IPMW for dealing with non‐monotone patterns; that is, our implementation of IPMW assumed that missing data were monotonic throughout. Recent developments on IPW have considered extensions to non‐monotone missing data patterns, but it remains unclear whether these can be incorporated into MSMs.^28^

The study has some limitations. This article focuses on IPW and MI, which are two general missing data approaches that are likely to be valid across a wide range of missing data patterns and mechanisms.^29^ A recent study^10^ has considered other methods for addressing missing data in settings with time varying confounding, such as “last observation carried forward” and “missingness pattern approach.” However, as the study highlights, these methods may be valid only in a limited number of missing data mechanisms and patterns, and hence were not considered in our article.

For the purposes of this study, the simulations only considered scenarios where data were MAR, as both IPW and MI are expected to be valid under the MAR assumption. If the probability of the outcome and confounder data being observed depends on unobserved values, that is, data are missing not at random (MNAR), then both IPW and MI are likely to be biased. In MNAR settings, the MI approach may provide a more flexible framework to conduct sensitivity analysis to potential departures from the MAR assumption.^30^, ^31^

Another limitation of our study was the simplification of the time‐varying causal pathways, as proposed in Figure 1. The causal mechanisms could be expanded in two ways: (1) by allowing longer‐term causal relationships between the variables; for example, impact of the treatment on more distant (future) measurements of the time‐varying confounder and outcome. (2) by considering more complex causal relationships. This could include settings where there is an effect modification by time‐varying confounding, or reverse causal pathways.^32^ Extensions to the time‐varying causal pathways are likely to have direct implications for the specification of the MSMs, but it is unclear how this would affect the choice of the missing data approach. This constitutes an interesting avenue for future research.

In conclusion, MI seems to be a more appropriate method for handling missing data in the estimation of time‐varying treatments in CER. In this context, missing data tend to permeate both outcomes and confounders, which creates difficulties for the IPW because it requires several sets of weights to be estimated and combined. CER studies are also typically faced with non‐monotone missing data patterns and large proportions of missing data, in which scenarios the relative advantages of MI vs IPW tend to be more pronounced.

## Supporting information


**Data S1.** Supplementary material.

## Data Availability

Data sharing is not applicable to this article as no new data were created or analyzed in this study.

## References

[1] Hernan MA . Methods of public Health Research—strengthening causal inference from observational data. N Engl J Med. 2021;385:1345‐1348. doi:10.1056/NEJMp2113319 34596980

[2] Bullement A , Podkonjak T , Robinson MJ , et al. Real‐world evidence use in assessments of cancer drugs by NICE. Int J Technol Assess Health Care. 2020;1‐7:388‐394. doi:10.1017/S0266462320000434 32646531

[3] Griffiths EA , Macaulay R , Vadlamudi NK , Uddin J , Samuels ER . The role of noncomparative evidence in health technology assessment decisions. Value Health. 2017;20:1245‐1251. doi:10.1016/j.jval.2017.06.015 29241883

[4] Makady A , van Veelen A , Jonsson P , et al. Using real‐world data in health technology assessment (HTA) practice: a comparative study of five HTA agencies. Pharmacoeconomics. 2018;36:359‐368. doi:10.1007/s40273-017-0596-z 29214389 PMC5834594

[5] Clare PJ , Dobbins TA , Mattick RP . Causal models adjusting for time‐varying confounding‐a systematic review of the literature. Int J Epidemiol. 2019;48:254‐265. doi:10.1093/ije/dyy218 30358847

[6] Daniel RM , Cousens SN , De Stavola BL , Kenward MG , Sterne JA . Methods for dealing with time‐dependent confounding. Stat Med. 2013;32:1584‐1618. doi:10.1002/sim.5686 23208861

[7] Robins JM , Hernan MA , Brumback B . Marginal structural models and causal inference in epidemiology. Epidemiology. 2000;11:550‐560. 10955408 10.1097/00001648-200009000-00011

[8] Bell ML , Fiero M , Horton NJ , Hsu CH . Handling missing data in RCTs; a review of the top medical journals. BMC Med Res Methodol. 2014;14:118. doi:10.1186/1471-2288-14-118 25407057 PMC4247714

[9] Leurent B , Gomes M , Carpenter JR . Missing data in trial‐based cost‐effectiveness analysis: an incomplete journey. Health Econ. 2018;27:1024‐1040. doi:10.1002/hec.3654 29573044 PMC5947820

[10] Leyrat C , Carpenter JR , Bailly S , Williamson EJ . Common methods for handling missing data in marginal structural models: what works and why. Am J Epidemiol. 2021;190:663‐672. doi:10.1093/aje/kwaa225 33057574 PMC8631064

[11] Liu SH , Chrysanthopoulou SA , Chang Q , Hunnicutt JN , Lapane KL . Missing data in marginal structural models: a plasmode simulation study comparing multiple imputation and inverse probability weighting. Med Care. 2019;57:237‐243. doi:10.1097/MLR.0000000000001063 30664611 PMC6436551

[12] Moodie EE , Delaney JA , Lefebvre G , Platt RW . Missing confounding data in marginal structural models: a comparison of inverse probability weighting and multiple imputation. Int J Biostat. 2008;4:13. doi:10.2202/1557-4679.1106 22462119

[13] Vourli G , Touloumi G . Performance of the marginal structural models under various scenarios of incomplete marker's values: a simulation study. Biom J. 2015;57:254‐270. doi:10.1002/bimj.201300159 25352223

[14] Bell ML , Fairclough DL . Practical and statistical issues in missing data for longitudinal patient‐reported outcomes. Stat Methods Med Res. 2014;23:440‐459. doi:10.1177/0962280213476378 23427225

[15] Fielding S , Fayers PM , Ramsay CR . Investigating the missing data mechanism in quality of life outcomes: a comparison of approaches. Health Qual Life Outcomes. 2009;7:57. doi:10.1186/1477-7525-7-57 19545408 PMC2711047

[16] Stevenson M , Archer R , Tosh J , et al. Adalimumab, etanercept, infliximab, certolizumab pegol, golimumab, tocilizumab and abatacept for the treatment of rheumatoid arthritis not previously treated with disease‐modifying antirheumatic drugs and after the failure of conventional disease‐modifying antirheumatic drugs only: systematic review and economic evaluation. Health Technol Assess. 2016;20:1‐610. doi:10.3310/hta20350 PMC4867425 27140438

[17] Wolfe F , Michaud K . The National Data Bank for rheumatic diseases: a multi‐registry rheumatic disease data bank. Rheumatology (Oxford). 2011;50:16‐24. doi:10.1093/rheumatology/keq155 20566735

[18] Rubin DB . Multiple Imputation for Nonresponse in Surveys. New York: Wiley; 1987.

[19] White IR , Royston P , Wood AM . Multiple imputation using chained equations: issues and guidance for practice. Stat Med. 2011;30:377‐399. doi:10.1002/Sim.4067 21225900

[20] Lee KJ , Carlin JB . Multiple imputation in the presence of non‐normal data. Stat Med. 2017;36:606‐617. doi:10.1002/sim.7173 27862164

[21] Morris TP , White IR , Royston P . Tuning multiple imputation by predictive mean matching and local residual draws. BMC Med Res Methodol. 2014;14:75. doi:10.1186/1471-2288-14-75 24903709 PMC4051964

[22] Morris TP , White IR , Crowther MJ . Using simulation studies to evaluate statistical methods. Stat Med. 2019;38:2074‐2102. doi:10.1002/sim.8086 30652356 PMC6492164

[23] Lenis D , Ackerman B , Stuart EA . Measuring model misspecification: application to propensity score methods with complex survey data. Comput Stat Data Anal. 2018;128:48‐57. doi:10.1016/j.csda.2018.05.003 29988991 PMC6034692

[24] Leyrat C , Seaman SR , White IR , et al. Propensity score analysis with partially observed covariates: how should multiple imputation be used? Stat Methods Med Res. 2019;28:3‐19. doi:10.1177/0962280217713032 28573919 PMC6313366

[25] Kreif N , Sofrygin O , Schmittdiel JA , et al. Exploiting nonsystematic covariate monitoring to broaden the scope of evidence about the causal effects of adaptive treatment strategies. Biometrics. 2021;77:329‐342. doi:10.1111/biom.13271 32297311

[26] Mojaverian N , Moodie EE , Bliu A , Klein MB . The impact of sparse follow‐up on marginal structural models for time‐to‐event data. Am J Epidemiol. 2015;182:1047‐1055. doi:10.1093/aje/kwv152 26589708 PMC4675663

[27] Seaman SR , White IR , Copas AJ , Li L . Combining multiple imputation and inverse‐probability weighting. Biometrics. 2012;68:129‐137. doi:10.1111/j.1541-0420.2011.01666.x 22050039 PMC3412287

[28] Sun B , Tchetgen Tchetgen EJ . On inverse probability weighting for nonmonotone missing at random data. J Am Stat Assoc. 2018;113:369‐379. doi:10.1080/01621459.2016.1256814 30034062 PMC6051732

[29] Molenberghs G , Fitzmaurice GM , Kenward MG , Tsiatis AA , Verbeke G . Handbook of Missing Data Methodology. New York: Chapman and Hall/CRC; 2015.

[30] Carpenter JR , Smuk M . Missing data: a statistical framework for practice. Biom J. 2021;63:915‐947. doi:10.1002/bimj.202000196 33624862 PMC7615108

[31] Carpenter J , Kenward M . Multiple Imputation and its Application. New York: Wiley; 2013.

[32] Newsome SJ , Keogh RH , Daniel RM . Estimating long‐term treatment effects in observational data: a comparison of the performance of different methods under real‐world uncertainty. Stat Med. 2018;37:2367‐2390. doi:10.1002/sim.7664 29671915 PMC6001810

