# Supplementary material for: Addressing missing data in the estimation of time‐varying treatments in comparative effectiveness research
Source: Stat Med. 2023 Sep 19;42(27):5025–38. doi: 10.1002/sim.9899 (PMC10947135; doi:10.1002/sim.9899)
Supplement: Supplementary file 1 — Data S1. Supplementary material. [file SIM-42-5025-s001.docx]

**Supplementary material for “Addressing missing data in the estimation of time-varying treatments in comparative effectiveness research”**

by Juan Segura-Buisan, Clemence Leyrat, Manuel Gomes

**Appendix 1: Data Generation Process**

We generated 1000 datasets for each of the 12 main scenarios using the equations below.

${seed}_{i}=floor(unif\left( 1,0 \right)*1000+1000*(i-1))$ for $1\leq i\leq1000$

For each dataset, we first generated the baseline ($t=0$) values of all variables, assuming that the outcome and time-varying confounder were Normally distributed:

$$a=14+76*beta\left( 4.23, 2.7 \right)$$

$$s=1(unif\left( 0, 1 \right)<0.06)$$

$$X_{0}=norm(1.09+s*0.1+a*0.00001, 0.714)$$

$$A_{0}=0$$

$${Cum\_A}_{t}=0$$

$$Y_{0}=norm(0.842-X_{0}*0.101+s*0.062 + a*0.0001, 0.152)$$

$$C_{0}={{Mo}_{0}=Mc}_{0}=0$$

Where $a$ and $s$ are the time-constant confounders, $X$ is the time-varying confounders, $A$ is the treatment indicator, $Cum\_A$ is the cumulative treatment, $Y$ is the outcome, $C$ is the censoring indicator, $Mo$ and $Mc$ are the missingness models for the outcome and confounder, respectively. For the subsequent phases ($t=1,\ldots,4$) we used the formulae below:

$X_{t}=$n$orm($1.62 + 0.38*$X_{t-1}-0.7*A_{t-1}-1.32*Y_{t-1}+0.016*s+0.001*a, 0.716)$

$$A_{t}=1(unif\left( 0, 1 \right)<expit(-2.2+0.4*X_{t}+1.8*A_{t-1}+0.31*Y_{t-1}+0.041*s+0.025*a))$$

$${Cum\_A}_{t}={Cum\_A}_{t-1}+A_{t}$$

$$Y_{t}=norm(0.32-0.036*X_{t}+0.05*A_{t}+0.63*Y_{t-1}-0.01*s+0.0001*a, 0.144)$$

$$C_{t}=1(unif\left( 0, 1 \right)<(expit\left( -\beta_{C}-2.1*A_{t}+0.25*s+0.025*a \right)+C_{t-1}-\gamma_{C})$$

$${Mo}_{t}=1(unif\left( 0, 1 \right)<(expit\left( -\beta_{Mo}-1.1*A_{t}+0.8*s+0.045*a \right)+C_{t-1}-\gamma_{Mo})$$

$${Mc}_{t}=1(unif\left( 0, 1 \right)<(expit\left( -\beta_{Mc}-1.4*A_{t}+0.7*s+0.055*a \right)+C_{t-1}-\gamma_{Mc})$$

Skewed outcomes

For scenarios with skewed outcome data, we assumed the outcome followed a Gamma distribution:

$$Y_{0}=gamma\left( \frac{\left( 0.842-X_{0}*0.101+s*0.062+a*0.0001 \right)^{2}}{\lambda*{0.152}^{2}}, \right.$$

$$\left. \frac{\lambda*{0.152}^{2}}{0.842-X_{0}*0.101+s*0.062+a*0.0001} \right)$$

$$Y_{t}=gamma\left( \frac{\left( 0.32-0.036*X_{t}+0.05*A_{t}+0.63*Y_{t-1}-0.01*s+0.0001*a \right)^{2}}{\lambda*{0.144}^{2}}, \right.$$

$$\left. \frac{\lambda*{0.144}^{2}}{0.32-0.036*X_{t}+0.05*A_{t}+0.63*Y_{t-1}-0.01*s+0.0001*a} \right)$$

Model misspecification

For scenarios with misspecification, we assumed outcome and probability of treatment included an interaction term between $X$ and $s$:

$$Y_{0}=norm\left( 0.842-X_{0}*0.101+s*0.062 + a*0.0001-0.1*DoM*X_{0}*s, 0.152 \right)$$

$$Y_{t}=norm\left( 0.32-0.036*X_{t}+0.05*A_{t}+0.63*Y_{t-1}-0.01*s+0.0001*a-0.1*DoM*X_{0}*s, 0.144 \right)$$

$$C_{t}=1(unif\left( 0, 1 \right)<(expit\left( -\beta_{C}-2.1*A_{t}+0.25*s+0.025*a+0.2*DoM*X_{0}*s \right)+C_{t-1}-\gamma_{C})$$

$${Mo}_{t}=1(unif\left( 0, 1 \right)<(expit\left( -\beta_{Mo}-1.1*A_{t}+0.8*s+0.045*a+0.5*DoM*X_{0}*s \right)+C_{t-1}-\gamma_{Mo})$$

$${Mc}_{t}=1(unif\left( 0, 1 \right)<(expit\left( -\beta_{Mc}-1.4*A_{t}+0.7*s+0.055*a+0.5*DoM*X_{0}*s \right)+C_{t-1}-\gamma_{Mc})$$

Where $DoM$ stands for ‘Degree of Misspecification’ and was set to ‘low’ (equal to 0.2) and ‘high’ (equal to 1).

**Appendix 2: Simulated scenarios**

To simulate the different scenarios, we varied the parameters $\beta$ and $\gamma$ as described in table below.

| **Non-monotone missingness** | | **Monotone missingness** | **Level of missingness** | **Skewness** | $\boldsymbol{\beta}_{\boldsymbol{C}}$ | $\boldsymbol{\beta}_{\boldsymbol{Mo}}$ | $\boldsymbol{\beta}_{\boldsymbol{Mc}}$ | $\boldsymbol{\gamma}_{\boldsymbol{C}}$ | $\boldsymbol{\gamma}_{\boldsymbol{Mo}}$ | $\boldsymbol{\gamma}_{\boldsymbol{Mc}}$ | $\boldsymbol{\lambda}$ |
| --- | --- | --- | --- | --- | --- | --- | --- | --- | --- | --- | --- |
| **Outcome** | **Confounder** |  |  |  |  |  |  |  |  |  |  |
| Yes (20%) | No | No | Low | - | - | 2.3 | - | 1 | 0 | 1 | - |
| No | Yes (20%) | No | Low | - | - | - | 2.8 | 1 | 1 | 0 | - |
| Yes (10%) | Yes (10%) | No | Low | - | - | 3.2 | 3.7 | 1 | 0 | 0 | - |
| Yes (10%) | No | Yes (10%) | Low | - | 3.6 | 3.2 | - | 0 | 0 | 1 | - |
| No | Yes (10%) | Yes (10%) | Low | - | 3.6 | - | 3.7 | 0 | 1 | 0 | - |
| Yes (5%) | Yes (5%) | Yes (10%) | Low | - | 3.6 | 4.0 | 4.5 | 0 | 0 | 0 | - |
| Yes (40%) | No | No | High | - | - | 1.1 | - | 1 | 0 | 1 | - |
| No | Yes (40%) | No | High | - | - | - | 1.5 | 1 | 1 | 0 | - |
| Yes (25%) | Yes (25%) | No | High | - | - | 2.0 | 2.4 | 1 | 0 | 0 | - |
| Yes (25%) | No | Yes (25%) | High | - | 2.4 | 2.0 | - | 0 | 0 | 1 | - |
| No | Yes (25%) | Yes (25%) | High | - | 2.4 | - | 2.4 | 0 | 1 | 0 | - |
| Yes (12.5%) | Yes (12.5%) | Yes (25%) | High | - | 2.4 | 2.9 | 3.4 | 0 | 0 | 0 | - |
| Yes (5%) | Yes (5%) | Yes (10%) | Low | Low | 3.6 | 4.0 | 4.5 | 0 | 0 | 0 | 10 |
| Yes (12.5%) | Yes (12.5%) | Yes (25%) | High | Low | 2.4 | 2.9 | 3.4 | 0 | 0 | 0 | 10 |
| Yes (5%) | Yes (5%) | Yes (10%) | Low | High | 3.6 | 4.0 | 4.5 | 0 | 0 | 0 | 30 |
| Yes (12.5%) | Yes (12.5%) | Yes (25%) | High | High | 2.4 | 2.9 | 3.4 | 0 | 0 | 0 | 30 |

**Table A1** Simulated parameters for each scenario

**Note**: When there is more than one type of missingness in the same scenario, these may overlap. Total amount of missing data was about 20% (40%) in scenarios with ‘low’ (‘high’) level of missingness, respectively.

**Appendix 3: Simulation results**

**Table A2** Full results for the scenarios with a Normally distributed outcome, N=5,000

| **Outcome** | **Confounder** | **Monotone** | **Missingness** | **MI** | | | **IPW** | | |
| --- | --- | --- | --- | --- | --- | --- | --- | --- | --- |
|  |  |  |  | Bias (%) | rMSE | Empirical SE | Bias (%) | rMSE | Empirical SE |
| Yes | No | No | Low | 1.28 | 0.0105 | 0.0102 | 0.78 | 0.0161 | 0.0160 |
| No | Yes | No | Low | 1.20 | 0.0099 | 0.0096 | 1.58 | 0.0194 | 0.0192 |
| Yes | Yes | No | Low | 1.22 | 0.0101 | 0.0098 | 1.46 | 0.0164 | 0.0161 |
| Yes | No | Yes | Low | 2.12 | 0.0115 | 0.0107 | 1.38 | 0.0137 | 0.0135 |
| No | Yes | Yes | Low | 0.98 | 0.0106 | 0.0104 | 1.84 | 0.0148 | 0.0143 |
| Yes | Yes | Yes | Low | 1.46 | 0.0110 | 0.0106 | 1.68 | 0.0139 | 0.0135 |
| Yes | No | No | High | 1.10 | 0.0116 | 0.0114 | 1.03 | 0.0297 | 0.0297 |
| No | Yes | No | High | 1.17 | 0.0099 | 0.0097 | 2.33 | 0.0477 | 0.0475 |
| Yes | Yes | No | High | 1.19 | 0.0108 | 0.0105 | 1.19 | 0.0393 | 0.0392 |
| Yes | No | Yes | High | 3.01 | 0.0149 | 0.0137 | 1.44 | 0.0241 | 0.0240 |
| No | Yes | Yes | High | 0.63 | 0.0130 | 0.0129 | 2.22 | 0.0311 | 0.0308 |
| Yes | Yes | Yes | High | 1.42 | 0.0135 | 0.0132 | 2.31 | 0.0250 | 0.0246 |

**Note**: IPW: inverse probability weighting. MI: multiple imputation. rMSE: root mean squared error. The scenarios differed according to whether there was non-monotone (outcome, confounder, or both) or monotone missing data, across low and high levels of missingness. The ‘true’ long-term treatment effect was 0.2.

**Table A3** Full results for the scenarios with a Normally distributed outcome, N=1,000

| **Outcome** | **Confounder** | **Monotone** | **Missingness** | **MI** | | | **IPW** | | |
| --- | --- | --- | --- | --- | --- | --- | --- | --- | --- |
|  |  |  |  | Bias (%) | rMSE | Empirical SE | Bias (%) | rMSE | Empirical SE |
| Yes | No | No | Low | 1.05 | 0.0237 | 0.0236 | 0.89 | 0.0368 | 0.0368 |
| No | Yes | No | Low | 0.87 | 0.0221 | 0.0221 | 1.01 | 0.0411 | 0.0410 |
| Yes | Yes | No | Low | 1.02 | 0.0228 | 0.0228 | 1.17 | 0.0345 | 0.0344 |
| Yes | No | Yes | Low | 1.80 | 0.0256 | 0.0254 | 1.48 | 0.0315 | 0.0314 |
| No | Yes | Yes | Low | 0.69 | 0.0245 | 0.0244 | 1.47 | 0.0314 | 0.0313 |
| Yes | Yes | Yes | Low | 1.27 | 0.0251 | 0.0250 | 1.51 | 0.0306 | 0.0304 |
| Yes | No | No | High | 1.07 | 0.0264 | 0.0263 | 0.94 | 0.0688 | 0.0688 |
| No | Yes | No | High | 0.65 | 0.0223 | 0.0223 | -1.28 | 0.0842 | 0.0843 |
| Yes | Yes | No | High | 1.01 | 0.0240 | 0.0240 | -1.11 | 0.0756 | 0.757 |
| Yes | No | Yes | High | 2.90 | 0.0322 | 0.0317 | 1.57 | 0.0523 | 0.0522 |
| No | Yes | Yes | High | 0.35 | 0.0285 | 0.0285 | 0.78 | 0.0596 | 0.0596 |
| Yes | Yes | Yes | High | 1.36 | 0.0303 | 0.0302 | 0.94 | 0.0526 | 0.0526 |

**Note**: IPW: inverse probability weighting. MI: multiple imputation. rMSE: root mean squared error. The scenarios differed according to whether there was non-monotone (outcome, confounder, or both) or monotone missing data, across low and high levels of missingness. The ‘true’ long-term treatment effect was 0.2.

**Table A4** Full results for the scenarios with a skewed outcome.

| **Outcome** | **Confounder** | **Censoring** | **Missingness** | **Skewness** | **MI (linear regression)** | | | **MI (PMM)** | | | **IPW** | | |
| --- | --- | --- | --- | --- | --- | --- | --- | --- | --- | --- | --- | --- | --- |
|  |  |  |  |  | Bias (%) | rMSE | Empirical SE | Bias (%) | rMSE | Empirical SE | Bias (%) | rMSE | Empirical SE |
| Yes | Yes | Yes | Low | Low | 3.85 | 0.0350 | 0.0342 | 3.71 | 0.0350 | 0.0342 | 1.85 | 0.0438 | 0.0437 |
| Yes | Yes | Yes | High | Low | 6.74 | 0.0436 | 0.0415 | 5.94 | 0.0429 | 0.0412 | 3.20 | 0.0775 | 0.0773 |
| Yes | Yes | Yes | Low | High | 3.69 | 0.0736 | 0.0733 | 2.28 | 0.0736 | 0.0734 | -0.65 | 0.0818 | 0.0818 |
| Yes | Yes | Yes | High | High | 7.64 | 0.0867 | 0.0853 | 3.05 | 0.0862 | 0.0861 | 1.64 | 0.1361 | 0.1362 |

**Note**: IPW: inverse probability weighting. MI: multiple imputation. PMM: predictive mean matching. rMSE: root mean squared error. All scenarios included both non-monotone (outcome, confounder, or both) and monotone missing data, but differed according to low and high levels of missingness and skewness. The ‘true’ average treatment effect was 0.2.

**Table A5** Full results for the scenarios with misspecification.

| **Outcome** | **Confounder** | **Censoring** | **Missingness** | **Misspec.** | **MI** | | | **IPW** | | |
| --- | --- | --- | --- | --- | --- | --- | --- | --- | --- | --- |
|  |  |  |  |  | Bias (%) | rMSE | Empirical SE | Bias (%) | rMSE | Empirical SE |
| Yes | Yes | Yes | Low | Low | 4.64 | 0.0142 | 0.0108 | 3.47 | 0.0153 | 0.0137 |
| Yes | Yes | Yes | High | Low | 3.99 | 0.0156 | 0.0134 | 3.24 | 0.0256 | 0.0248 |
| Yes | Yes | Yes | Low | High | 6.14 | 0.0172 | 0.0122 | 1.34 | 0.0150 | 0.0148 |
| Yes | Yes | Yes | High | High | 3.42 | 0.0158 | 0.0142 | 1.32 | 0.0264 | 0.0236 |

**Note**: IPW: inverse probability weighting. MI: multiple imputation. rMSE: root mean squared error. All scenarios included non-monotone (both outcome and confounder) and monotone missing data but differed according to low and high levels of missingness and misspecification. The ‘true’ average treatment effect was 0.2.
